# Afferent signalling from the acid-challenged rat stomach is inhibited and gastric acid elimination is enhanced by lafutidine

**DOI:** 10.1186/1471-230X-9-40

**Published:** 2009-06-02

**Authors:** Martin E Edelsbrunner, Motoko Nakano, Peter Holzer

**Affiliations:** 1 Research Unit of Translational Neurogastroenterology, Institute of Experimental and Clinical Pharmacology, Medical University of Graz, Universitätsplatz 4, A-8010 Graz, Austria; 2 Optimal Medication Research Laboratory, Tokushima Research Center, Taiho Pharmaceutical Co. Ltd., 224-2 Ebisuno, Hiraishi, Kawauchi-cho, Tokushima-City, 771-0194 Japan

## Abstract

**Background:**

Lafutidine is a histamine H_2 _receptor antagonist, the gastroprotective effect of which is related to its antisecretory activity and its ability to activate a sensory neuron-dependent mechanism of defence. The present study investigated whether intragastric administration of lafutidine (10 and 30 mg/kg) modifies vagal afferent signalling, mucosal injury, intragastric acidity and gastric emptying after gastric acid challenge.

**Methods:**

Adult rats were treated with vehicle, lafutidine (10 – 30 mg/kg) or cimetidine (10 mg/kg), and 30 min later their stomachs were exposed to exogenous HCl (0.25 M). During the period of 2 h post-HCl, intragastric pH, gastric volume, gastric acidity and extent of macroscopic gastric mucosal injury were determined and the activation of neurons in the brainstem was visualized by c-Fos immunocytochemistry.

**Results:**

Gastric acid challenge enhanced the expression of c-Fos in the nucleus tractus solitarii but caused only minimal damage to the gastric mucosa. Lafutidine reduced the HCl-evoked expression of c-Fos in the NTS and elevated the intragastric pH following intragastric administration of excess HCl. Further analysis showed that the gastroprotective effect of lafutidine against excess acid was delayed and went in parallel with facilitation of gastric emptying, measured indirectly via gastric volume changes, and a reduction of gastric acidity. The H_2 _receptor antagonist cimetidine had similar but weaker effects.

**Conclusion:**

These observations indicate that lafutidine inhibits the vagal afferent signalling of a gastric acid insult, which may reflect an inhibitory action on acid-induced gastric pain. The ability of lafutidine to decrease intragastric acidity following exposure to excess HCl cannot be explained by its antisecretory activity but appears to reflect dilution and/or emptying of the acid load into the duodenum. This profile of actions emphasizes the notion that H_2 _receptor antagonists can protect the gastric mucosa from acid injury independently of their ability to suppress gastric acid secretion.

## Background

Exposure of the gastric mucosa of conscious rats to excess HCl (0.25 M) is signalled, via vagal afferents, to the nucleus tractus solitarii (NTS) in the brainstem, where neuronal activation can be visualized by c-fos mRNA in-situ hybridization and c-Fos immunocytochemistry [1,2]. Analysis of the behavioural reactions to gastric acid challenge indicates that vagal afferent neurons play an important role in gastric acid nociception [3]. The vagal afferent signalling of gastric acid challenge is known to be inhibited by morphine [1], by a combination of glutamate NMDA and tachykinin NK_1 _and NK_2 _receptor antagonists [4] and by antisecretory agents such as cimetidine and omeprazole [2].

Like cimetidine, lafutidine is a histamine H_2 _receptor antagonist which has been shown to protect from acid-related gastric injury in rodents [5-8]. In addition, there is evidence that the gastroprotective action of lafutidine involves release of neuropeptides from afferent nerve endings in the stomach [7,8]. In view of this pharmacological profile the question arose as to whether lafutidine would be able to modify vagal afferent signalling of gastric acid challenge to the brainstem. Therefore, the first aim of this study was to test the effect of lafutidine on gastric acid-evoked expression of c-Fos in the rat brainstem. In addition, the gastric pH was recorded and the degree of macroscopic gastric injury quantified.

In the course of these experiments it was discovered that lafutidine facilitates the removal of the exogenous acid load from the stomach. Since secretion of endogenous gastric acid is suppressed by an exogenous acid load, the action of lafutidine to raise intragastric pH cannot be explained by its antisecretory activity due to histamine H_2 _receptor blockade. As a consequence, this effect of lafutidine is likely to reflect dilution and/or emptying of the acid load into the duodenum. This reasoning is supported by the observation that exposure of the stomach to excess acid inhibits gastric emptying and causes fluid secretion [2,9]. The second aim of this study was hence to explore whether lafutidine modifies the time course of gastric injury, gastric volume and gastric acidity following an exogenous acid load and whether any influence of lafutidine on these parameters is shared by cimetidine.

## Methods

### Animals

The study was carried out with female age-matched Sprague-Dawley rats (Division of Laboratory Animal Science and Genetics, Department of Biomedical Research, Medical University of Vienna, Himberg, Austria) weighing 170 – 250 g. The animals were housed in groups of 3 per cage under controlled temperature (21°C) and a 12 h light/dark cycle (lights on at 6:00, lights off at 18:00). All experiments were approved by an Ethical Committee at the Federal Ministry of Science and Research and conducted according to the Directive of the European Communities Council of 24 November 1986 (86/609/EEC). The experiments were designed in such a way that the number of animals used and their suffering was minimized.

### Experimental protocols

Two studies were carried out. Seven days before the gastric acid loading experiment, the animals were randomly assigned to treatment groups using a randomization software (Randomizer for Clinical Trials 1.8.0, Institute of Medical Informatics, Statistics and Documentation, Medical University Graz, Austria). On the day before the acid loading experiment, the rats were deprived of food and fasted for 18 h to ensure that the stomach was empty, but had free access to water. In the first study, the effects of two doses of lafutidine (10 and 30 mg/kg) on gastric acid-evoked c-Fos expression in the brainstem of conscious rats was examined along with gastric acidity and injury. Lafutidine or its vehicle (0.5% carboxymethyl cellulose) was administered perorally 30 min before the stomach was exposed to a noxious concentration of acid (0.25 M HCl) by gavage. After the intragastric treatment the animals were no longer allowed to drink until recording of the experimental parameters. Two hours after gastric acid loading the number of c-Fos-immunoreactive neurons in the brainstem, intragastric pH and the extent of macrosopic gastric mucosal injury were determined.

In the second experiment, the effects of a single dose of lafutidine (30 mg/kg) or cimetidine (10 mg/kg) on the time course of gastric volume, acidity and injury after exposure to an exogenous acid load were examined. The drugs as well as their vehicle were administered intragastrically by gavage 30 min before the stomach was exposed to HCl. After the intragastric treatment the animals were no longer allowed to drink until recording of the experimental parameters. Gastric volume, acidity and injury were quantified at 3 time points after administration of the intragastric acid load: 0, 60 and 120 min. All parameters were recorded in the same experiments.

### Lafutidine and cimetidine treatments

Lafutidine (Taiho, Tokyo, Japan) and cimetidine (Sigma, Vienna, Austria) were suspended in carboxymethyl cellulose (0.5%) at a concentration of 2 and 6 mg/ml (lafutidine) and 2 mg/ml (cimetidine). Lafutidine (10 and 30 mg/kg), cimetidine (10 mg/kg) or its vehicle was administered perorally by gavage at a volume of 5 ml/kg 30 min before the stomach was exposed to HCl. The intragastric administration was carried out with a soft infant feeding tube (outer diameter 2.6 mm; SIMS Portex, Hythe, United Kingdom).

### Gastric acid loading

HCl (0.25 M) was administered intragastrically at a volume of 10 ml/kg through the same soft infant feeding tube that was used for administration of lafutidine. After this intragastric acid loading the animals were no longer allowed to drink until recording of the experimental parameters.

### c-Fos immunocytochemistry

c-Fos-like immunoreactivity was visualized as described previously [2,10]. Two hours after intragastric acid loading, the rats were euthanized by intraperitoneal injection of an overdose of pentobarbital (200 mg/kg). Following euthanasia, the animals were transcardially perfused with buffered paraformaldehyde (4%, 75 ml) while the descending aorta was clamped. The brainstems were removed and postfixed overnight in buffered paraformaldehyde (4%) at 4°C. Then the tissues were cryoprotected for 48 h in 20% sucrose at 4°C, frozen by immersion in methylbutane on dry ice and stored at -70°C until use. Serial coronal sections of 40 μm thickness were cut from the brainstem over the whole length of the area postrema (AP) with a cryostat.

Immunocytochemistry was performed with free-floating sections which first were washed once in 0.1 M phosphate-buffered saline (PBS), then washed twice in washing buffer (WB; 0.1 M PBS with 0.3% Triton X 100), and incubated in 0.3% H_2_O_2 _for 30 min. After three further washes (each for 10 min in WB), the tissues were incubated with the primary antibody (rabbit polyclonal anti-c-Fos, 1:20,000, Santa Cruz Biotech, Santa Cruz, California, USA) for 40 h at 4°C. This antibody was dissolved in 0.1 M PBS containing 0.3% Triton X 100, 1% bovine serum albumin and 2.5% goat serum. Afterwards the sections were washed three times in WB and incubated for 45 min in a solution containing the biotinylated secondary antibody (goat anti-rabbit IgG, Vectastain Elite Kit, Vector Laboratories, Burlingame, California, USA). After three other washes in WB they were incubated for 1 h in avidin-biotin complex (Vectastain Elite Kit). The tissues were rinsed afterwards and developed with 3,3-diaminobenzidine (DAB) substrate (Vectastain Elite Kit) intensified with nickel sulfate for 200 s. Subsequently the sections were mounted on gelatin-covered slides, air-dried and cleared in xylol (100%). The slides were coverslipped with Entellan (Merck, Darmstadt, Germany). To control for the specificity of the anti-c-Fos antibody signal, a c-Fos blocking peptide (Santa Cruz Biotech) was added to the primary antibody dilution.

The immunocytochemically processed brainstem sections were examined with a light microscope (Axiophot, Zeiss, Oberkochen, Germany) coupled to a computerized image analysis system (MCID-M2, version 3.0, Rev 1.1, Imaging Research Inc., Brock University, St. Catharines, Ontario, Canada). The sections were coded such that the examiner did not know which treatment group they came from. Four sections from the brainstem of each animal were analysed, and all c-Fos-positive cells were randomly counted on one side of the NTS and AP. In order to avoid that the same cells were counted twice, only every second section was taken for analysis. All counts in each section of each animal were averaged to give the number of c-Fos-positive cells in the NTS and AP of that animal. These average values from each animal were then used to calculate the mean number of c-Fos-positive cells per section in the unilateral NTS and AP of each experimental group.

### Determination of gastric volume

The rats were euthanized by intraperitoneal injection of an overdose of pentobarbital (200 mg/kg) immediately (0 min), 60 or 120 min after intragastric acid loading. Following exposure of the stomach by a midline laparotomy, the cardia and pylorus were clamped. The stomach was excised and weighed (weight 1). After collecting its content in a vial, the stomach was re-weighed (weight 2). The volume of the gastric content was calculated by the difference weight 2 – weight 1 and expressed relative to the body weight (g/kg body weight). In addition, the gastric volume recovered 60 and 120 min after gastric acid loading was also expressed relative to the volume measured immediately (0 min) after gastric acid loading.

### Intragastric pH

Following euthanasia, the abdomen was opened by a midline laparotomy. The stomach was excised and opened, and the intragastric pH was determined with a pH meter that was fitted with a Micro Line pH electrode (ThermoOrion, New Hyde Park, NY, USA) and calibrated with standard buffers of pH 4.01, 7.00 and 10.00.

### Titration of gastric contents

The gastric contents were emptied into tubes, briefly centrifuged at 5,600 × g (10,000 rpm) and diluted 1:10, 1:25 or 1:50 in 5 ml distilled water, the dilution rate depending on the volume of the gastric contents that were recovered. If no gastric juice was recovered, the appropriate amount of chyme was used. Titration was performed with 0.1 M NaOH on a Titroline Alpha Titration Apparatus (Schott-Geräte GmbH, Hofheim, Germany) until the endpoint of pH = 7.0 was reached. The amount of acid equivalents present in the stomach was calculated as mmol, and gastric acidity measured 60 and 120 min after gastric acid loading was also expressed relative to the acidity measured immediately (0 min) after gastric acid loading.

### Gastric injury

The integrity of the gastric mucosa was examined at the macroscopic level. To quantify macroscopically visible damage, the stomach was pinned flat on a silicon elastomer-coated plate and covered with PBS. The stomach was photographed, the image transferred to a personal computer, and macroscopic gastric injury assessed with computerized planimetry by an observer who was unaware of the experimental treatment [1]. The mucosal area covered by visible haemorrhagic damage was expressed as a percentage of the total area of the glandular mucosa.

### Statistics

Statistical evaluation of the results was performed on SPSS 15.0 (SPSS Inc., Chicago, IL, USA) with one-way analysis of variance (ANOVA). These tests were carried out despite the fact that the data of some experimental groups did not meet the criterion of normal distribution, because this limitation was explained by the limited number of animals per group and because there was no reason to assume a non-normal distribution. The homogeneity of variances was assessed with the Levene test. If a significant interaction between the test factors was found, post-hoc analysis of group differences was made with the Tukey HSD (honestly significant difference) test or, in case of inhomogeneity of variances, with the Games-Howell test. All data are presented as means ± SEM, n referring to the number of rats in each group. In view of the exploratory nature of the study, probability values ≤ 0.1 [11,12] were regarded as statistically significant.

## Results

### Study 1: Effects of lafutidine on c-Fos expression in the brainstem, intragastric pH and gastric mucosal injury after intragastric acid loading

**Figure 1:**
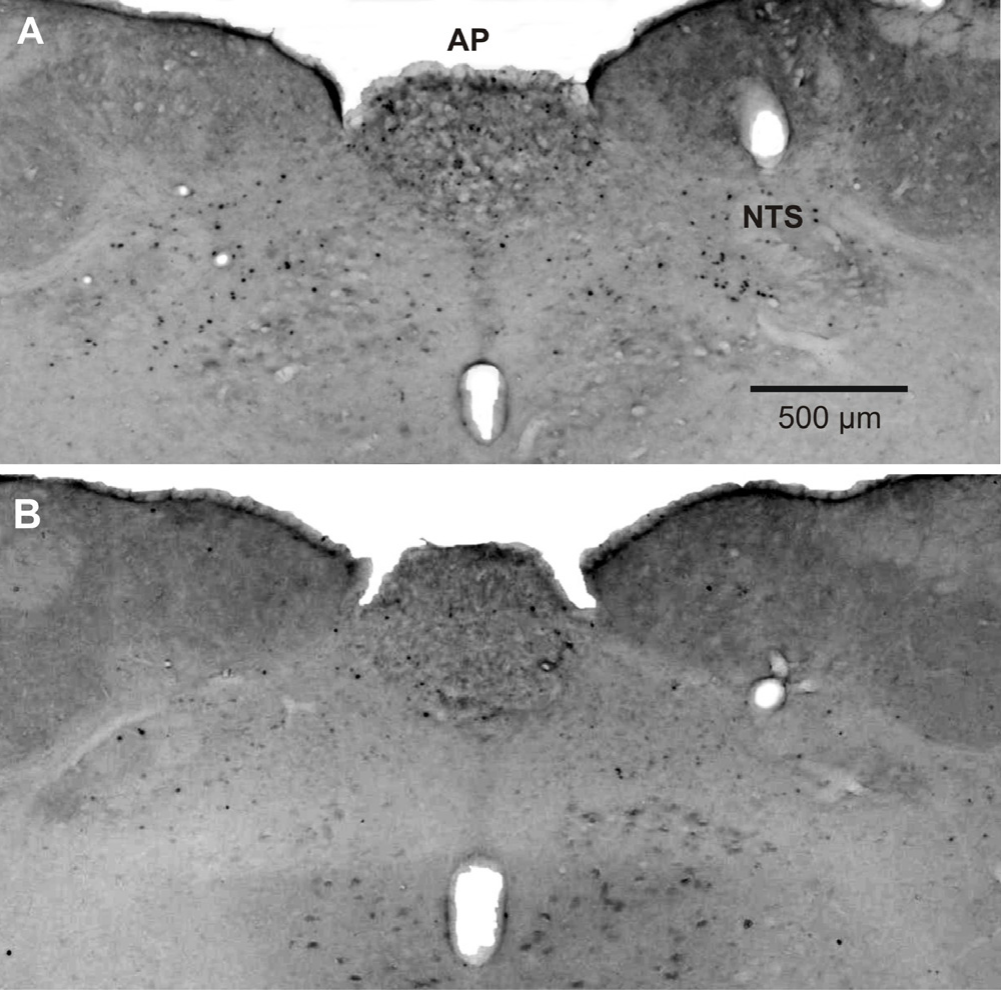
**Gastric acid-evoked expression of c-Fos in the nucleus tractus solitarii (NTS) and area postrema (AP) in a rat treated with vehicle (A) and another rat treated with lafutidine (B)**. Lafutidine (30 mg/kg) or its vehicle (0.5% carboxymethyl cellulose) was administered perorally 30 min before the stomach was exposed to an exogenous acid load (0.25 M HCl). Two hours post-HCl c-Fos-positive cells in the brainstem were visualized by immunocytochemistry.

**Figure 2:**
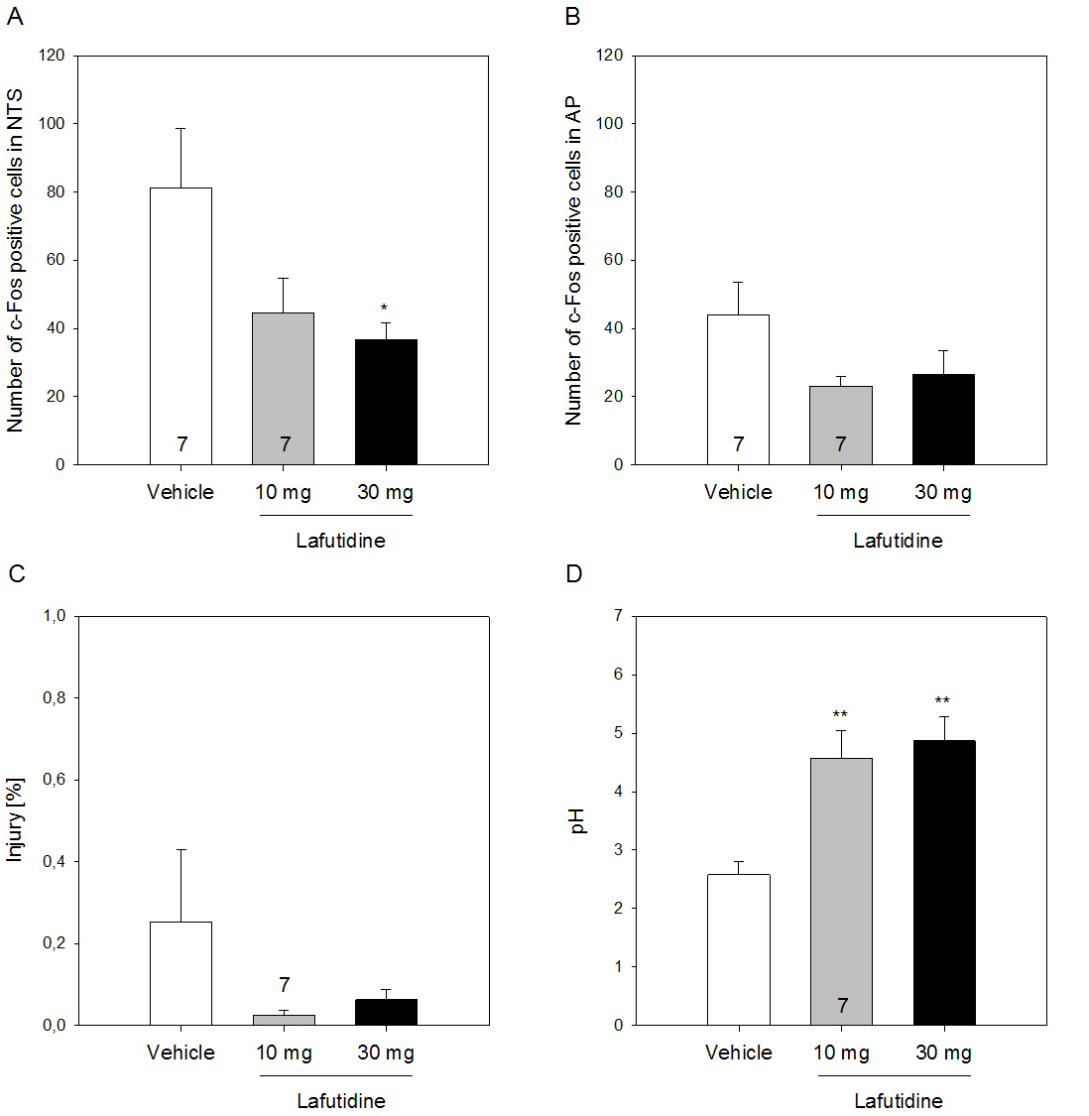
**Effect of lafutidine (10 and 30 mg/kg), relative to vehicle, on the number of c-Fos positive cells in (A) the nucleus tractus solitarii (NTS) and (B) area postrema (AP), (C) macroscopic gastric mucosal injury (expressed as a percentage of the area of the glandular mucosa) and (D) intragastric pH measured 2 h after exposure of the stomach to HCl (0.25 M)**. Lafutidine or vehicle was administered by gastric gavage 30 min before administration of HCl. The values represent means + SEM, n = 8 if not stated otherwise. * P ≤ 0.1, ** P ≤ 0.05 versus vehicle.

Exposure of the rat gastric mucosa to HCl (0.25 M) enhanced the expression of c-Fos in the NTS and AP to a significant extent (Figures 1A, B and 2A, B) but, relative to saline, did not cause any significant gastric mucosal damage (Danzer et al. 2004). A similar observation was made in the present study, given that on average less than 0.2% of the glandular mucosa presented with macroscopic abnormalities, mostly petechiae (Figure 2C). While the pH of the HCl solution administered intragastrically was 0.51, the pH in the gastric juice 2 h after HCl challenge had risen to more than 2.5 (Figure 2D).

Following intragastric administration of lafutidine (10 and 30 mg/kg), the area of gastric injury was nominally reduced but this effect did not reach statistical significance (Figure 2C). In contrast, intragastric pH was increased by either dose of lafutidine (Figure 2D) to a significant extent as revealed by one-way ANOVA (F_(2,20) _= 11.08, P < 0.001) and the post-hoc test. The acid-evoked expression of c-Fos in the NTS (Figures 1A, B and 2A) was reduced by lafutidine (10 and 30 mg/kg), and one-way ANOVA disclosed this effect to be statistically significant (F_(2,19) _= 4.30, P = 0.029). To the contrary, the ability of lafutidine to attenuate acid-evoked expression of c-Fos in the AP did not reach statistical significance (Figures 1A, B and 2B).

### Study 2: Effects of lafutidine on the time course of gastric volume, acidity and injury after intragastric acid loading

**Figure 3:**
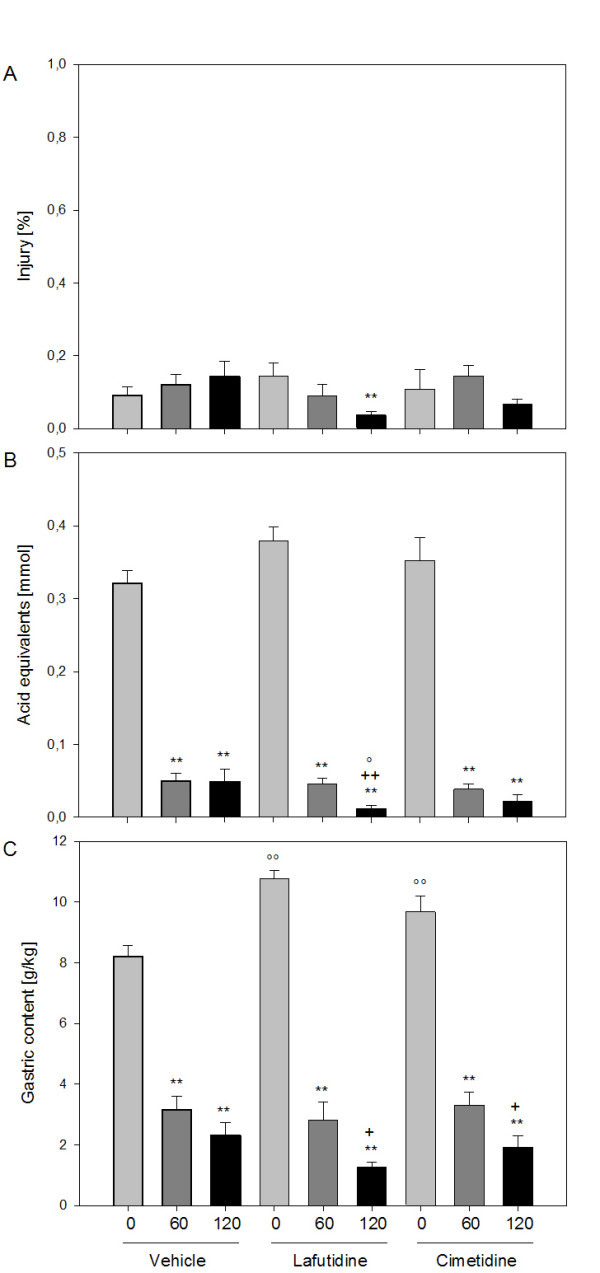
**Effect of lafutidine (30 mg/kg) and cimetidine (10 mg/kg), relative to vehicle, on (A) macroscopic gastric mucosal injury (expressed as a percentage of the area of the glandular mucosa), (B) intragastric acidity (acid equivalents in mmol) and (C) weight of gastric content (g/kg body weight) measured immediately (0 min), 60 and 120 min after exposure of the stomach to HCl (0.25 M)**. Lafutidine, cimetidine or vehicle was administered by gastric gavage 30 min before administration of HCl. The values represent means + SEM, n = 8. ** P ≤ 0.05 versus time 0 min under the same treatment, + P ≤ 0.1, ++ P ≤ 0.05 versus time 60 min under the same treatment, °P ≤ 0.1, °°P ≤ 0.05 versus vehicle at the same time point post-HCl.

As in study 1, challenge of the stomach with 0.25 M HCl caused minor macroscopic damage which on average covered less than 0.2% of the area of the glandular mucosa (Figure 3A). The extent of damage did not significantly differ between the time points 0, 60 and 120 min post-HCl in vehicle- and cimetidine-treated rats. In lafutidine-treated rats, however, the extent of damage 120 min after acid challenge was significantly smaller than immediately after gastric acid loading (Figure 3A).

**Figure 4:**
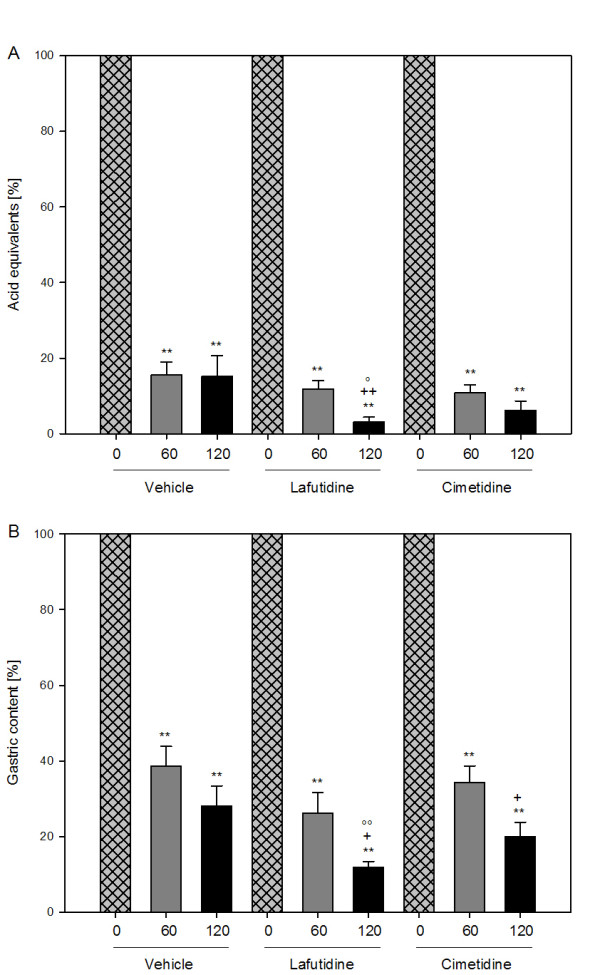
**Effect of lafutidine (30 mg/kg) and cimetidine (10 mg/kg), relative to vehicle, on (A) intragastric acidity and (B) weight of gastric content measured 60 and 120 min after exposure of the stomach to HCl (0.25 M) and expressed as a percentage of the respective values measured immediately after gastric acid loading (0 min)**. Lafutidine, cimetidine or vehicle was administered by gastric gavage 30 min before administration of HCl. The values represent means + SEM, n = 8. ** P ≤ 0.05 versus time 0 min under the same treatment, + P ≤ 0.1, ++ P ≤ 0.05 versus time 60 min under the same treatment, °P ≤ 0.1, °°P ≤ 0.05 versus vehicle at the same time point post-HCl.

The amount of acid equivalents present in the gastric lumen fell significantly over the 2 h interval post-HCl in all treatment groups (Figure 3B). Most of the drop in gastric acidity occurred within 60 min post-HCl, and there was no significant difference in the gastric acidity levels 0 and 60 min post-HCl between the three treatment groups. In lafutidine-treated rats there was a further significant drop of gastric acidity during the period 60 – 120 min post-HCl, a change that was not seen in vehicle- and cimetidine-treated rats (Figure 3B). The decrease in gastric acidity 120 min post-HCl in lafutidine-treated rats was significantly more marked than in vehicle-treated animals (Figure 3B). When gastric acidity measured 60 and 120 min post-HCl was expressed relative to the acidity measured immediately (0 min) after gastric acid loading, it was found that the drop of gastric acidity 120 min post-HCl seen in lafutidine-treated rats was most pronounced, compared with that seen in vehicle- and cimetidine-treated rats (Figure 4A).

The weight of the gastric content relative to the body weight (g/kg) was calculated as an indirect measure of gastric emptying. As shown in Figure 3C, the gastric content weight fell significantly over time in all treatment groups and 60 as well as 120 min post-HCl was significantly smaller than immediately after gastric acid loading. Lafutidine- and cimetidine-treated rats differed from vehicle-treated rats in two aspects. First, immediately after acid challenge the gastric content weight was significantly higher in lafutidine- and cimetidine-treated animals than in control rats (Figure 3C). Second, when measured 120 min post-HCl in lafutidine- and cimetidine-treated animals, this parameter was significantly lower than that measured 60 min post-HCl, whereas in vehicle-treated rats the gastric content weights recorded 60 and 120 min post-HCl were not significantly different from each other (Figure 3C).

Since the initial gastric volume in lafutidine- and cimetidine-treated animals was higher than in control rats (Figure 3C), the time course of gastric volume changes in the different treatment groups is difficult to compare with each other because it might be differentially influenced by the initial volume. For this reason, the gastric contents recovered 60 and 120 min post-HCl were also expressed as a percentage of the volume measured immediately (0 min) after gastric acid loading (Figure 4). In this way it was found that the decrease in gastric volume 120 min post-HCl in lafutidine-treated rats was more pronounced than in vehicle-treated rats (Figure 4B).

## Discussion

The major results of the current study can be summarized as follows. Lafutidine inhibits the vagal afferent signalling of a gastric acid insult, which raises the possibility that lafutidine inhibits acid-induced gastric pain. The ability of lafutidine to decrease intragastric acidity following exposure to excess HCl cannot be explained by its antisecretory activity and is likely to reflect dilution and/or emptying of the acid load into the duodenum.

Vagal afferent signalling of a gastric acid insult was visualized by c-Fos expression in the medullary brainstem, a standard method in functional neuroanatomy to delineate stimulus-evoked activation of neurons [13,14]. In this way it has previously been shown that exposure of the rat stomach to excess concentrations of HCl stimulates neurons in the brainstem [1,2]. The appearance of the c-Fos protein was measured 2 h post-HCl, given that the translation of c-fos mRNA into c-Fos protein reaches its maximum between 1 and 3 h post-stimulus [2]. We limited our analysis to the brainstem, because exposure of the rat stomach to HCl failed to induce any c-fos mRNA and c-Fos protein in the dorsal horn of the posterior thoracic spinal cord which receives gastric input via spinal afferent neurons [1,2]. These data and the ability of chronic bilateral subdiaphragmatic vagotomy to suppress gastric acid-evoked expression of c-fos mRNA [1] indicate that gastric challenge with HCl is signalled to the brainstem via vagal afferents.

The induction of c-fos mRNA and c-Fos protein in the NTS, the central projection area of vagal afferents, is related to the intragastrically administered HCl concentration [1,2]. A comparative analysis of the medullary c-Fos induction and gastric damage indicates that the afferent signalling of gastric acid challenge is not directly related to the formation of overt mucosal injury, since c-Fos expression in the NTS can be evoked by HCl concentrations (0.15 – 0.35 M) that do not induce any appreciable macroscopic lesions and cause little histological damage [1,2]. Because supraphysiological concentrations of HCl (0.15 M or higher) are required to induce c-Fos in the NTS, it has been inferred that only a massive increase in the proton gradient across the acid-tight gastric mucosal barrier is able to drive sufficient protons into the lamina propria where they can excite vagal afferent nerve fibres either directly or indirectly via neuroactive factors released in the tissue [2]. This experimental setup is thus thought to model pathophysiological circumstances where backdiffusion of luminal acid stimulates vagal afferents.

The data of the current study show that lafutidine administered intragastrically at doses (10 and 30 mg/kg) found previously to be gastroprotective [5,6,15] reduced the afferent signalling of a gastric acid insult to the NTS. This observation is consistent with the ability of another histamine H_2 _receptor antagonist, cimetidine, and the proton pump inhibitor omeprazole to reduce gastric acid-evoked expression of c-Fos in the rat brainstem [2]. Since the behavioural reactions to gastric acid challenge indicate that the c-Fos expression in the NTS is a correlate of gastric acid nociception [2,3] it can be proposed that lafutidine has an inhibitory effect on gastric chemonociception. This antinociceptive effect, however, is unlikely to be explained by the antisecretory activity of lafutidine, given that exposure of the gastric mucosa to excess exogenous acid such as 0.25 M HCl will by itself suppress endogenous acid secretion by a negative feedback mechanism [16-19]. As a consequence, two alternative explanations need to be envisaged.

One explanation that comes to mind is to assume that lafutidine, like cimetidine [2], interferes with vagal afferent pathways that signal acid challenge of the gastric mucosa to the brainstem. Lafutidine may do so (1) by inhibiting the stimulation of vagal afferent neurons by acid intruding the gastric mucosa, (2) by depressing signal conduction in vagal afferent neurons, (3) by blocking synaptic transmission between the central endings of vagal afferents and NTS neurons and/or (4) by reducing the excitability of the brainstem neurons expressing c-Fos in response to input from the stomach.

Which of these possibilities applies to the effects of lafutidine and cimetidine requires identification of the site of action where H_2 _receptor antagonists interfere with afferent vagal pathways. It has previously been shown that vagal afferent neurons that signal gastric acid challenge to the NTS are insensitive to the neurotoxic effect of capsaicin [1] and that the activation of this pathway by acid is independent of prostaglandins [20]. The vagal afferent system inhibited by lafutidine thus appears to be fundamentally different from that of capsaicin-sensitive spinal afferent neurons which are thought to mediate the ability of lafutidine to protect the gastrointestinal mucosa from injury [6-8,15,21-23]. While vagal afferents appear to be inhibited via a H_2 _receptor-dependent mechanism, capsaicin-sensitive spinal afferents are activated or sensitized by lafutidine, but not other H_2 _receptor antagonists, which results in the release of the protective messenger calcitonin gene-related peptide [6-8,15,21-23]. Prostaglandins may also play a role in the gastroprotective effect of lafutidine against stress [23].

Another explanation for the inhibitory effect of lafutidine on the gastric acid-evoked c-Fos expression in the brainstem takes into account that lafutidine significantly decreased the acidity of the exogenous gastric acid load. By whatever mechanism lafutidine facilitates the elimination of the gastric acid load, it will also diminish the stimulus for vagal afferent signalling to the NTS. Which of the two mechanisms – direct inhibition of the vagal afferent system or reduction of stimulus strength – is more relevant cannot be decided without electrophysiological characterization of the effect of H_2 _receptor antagonists on the vagal afferent system.

A direct analysis of the gastroprotective effect of lafutidine in the current study was out of scope because the gastric acid load used here (0.25 M HCl) induced only minimal gastric damage that was indistinguishable from that seen after intragastric administration of saline [2]. As a consequence, gastric acid-induced injury remained unaltered by lafutidine in study 1, although there was a tendency towards suppression of gastric lesion formation. Analysis of the time course of the effect of lafutidine in study 2, however, revealed that lafutidine, unlike vehicle, accelerated the recovery from HCl-induced gastric damage.

The gastroprotective potential of lafutidine is further envisaged from its effect to enhance intragastric pH and to reduce gastric acidity following exposure to an exogenous acid load. Since, as discussed above, endogenous gastric acid secretion is suppressed by an exogenous acid load as high as 0.25 M HCl, the action of lafutidine to decrease gastric acidity may be the result of dilution, neutralization or emptying of the acid load into the duodenum. It has previously been found that exposure of the stomach to supraphysiological concentrations of acid inhibits gastric emptying and causes fluid secretion [2,9], which in the current study was mirrored by the relatively slow decrease of the gastric volume over the 2 h interval post-HCl.

Lafutidine and cimetidine appeared to accelerate gastric emptying during the period 60 – 120 min post-HCl as indirectly deduced from a decrease in gastric volume that was more pronounced than in vehicle-treated animals. In contrast, the gastric volume immediately after gastric acid loading was significantly increased by lafutidine and cimetidine, which suggests that the secretion of gastric fluid was enhanced. The effect of lafutidine to reduce gastric acidity during the period of 60 – 120 min post-HCl coincided with its apparent effect to accelerate gastric emptying. Apart from this mechanism of action it is also conceivable that lafutidine facilitated gastric acid elimination by dilution with fluid and acid-neutralizing factors. The capacity of lafutidine to stimulate secretory processes other than acid secretion in the upper gut is supported by its effect to increase acid-stimulated duodenal bicarbonate secretion [7].

Cimetidine shared the effect of lafutidine to inhibit gastric acid-evoked expression of c-Fos in the NTS [2] and mimicked, in a qualitative manner, the effects of lafutidine to blunt gastric lesion formation, to decrease gastric acidity and to initially enhance and later reduce gastric volume following exposure to an exogenous acid load. The smaller effect of cimetidine, relative to that of cimetidine, is explained by its smaller potency as a H_2 _receptor antagonist [5].

## Conclusion

Lafutidine has been shown to inhibit gastric acid-evoked vagal nociceptive signalling and to decrease gastric acidity following an exogenous acid load. The antinociceptive effect of lafutidine may arise from an inhibitory effect on acid-sensitive vagal afferent pathways, whereas the ability of lafutidine to decrease the acidity of an exogenous acid load may result from accelerated gastric emptying and enhanced gastric fluid secretion. This profile of actions appears to be shared by cimetidine, which implies that histamine H_2 _receptor antagonists can protect the gastric mucosa from acid injury independently of their ability to suppress gastric acid secretion.

## Competing interests

This study was financially supported by Taiho Phamaceutical Co. Ltd. (Tokushima, Japan). Motoko Nakano is an employee of Taiho Phamaceutical Co. Ltd. (Tokushima, Japan). Martin E. Edelsbrunner and Peter Holzer declare that they have no competing interests.

## Authors' contributions

MEE perfomed the animal experiments including data acquisition and statistical analysis and drafted the manuscript together with PH. MN and PH designed and coordinated the study and were involved in the discussion and interpretation of the results. All authors read and approved the final manuscript.

## Pre-publication history

The pre-publication history for this paper can be accessed here:

http://www.biomedcentral.com/1471-230X/9/40/prepub
